# Case Report: Acute complete esophageal obstruction associated with intraesophageal coagulation of RuiNeng enteral nutrition formula

**DOI:** 10.3389/fnut.2026.1843691

**Published:** 2026-07-08

**Authors:** Yuqi Shen, Xinrui Jiang, Xiaoyu Cai, Zhen Wang

**Affiliations:** Department of Respiratory and Critical Care Medicine, The Third People's Hospital of Chengdu, Chengdu, Sichuan Province, China

**Keywords:** case report, enteral nutrition, esophageal bezoar, esophageal obstruction, formula coagulation, nasogastric tube

## Abstract

Enteral nutrition is widely used in critically ill patients and in patients with dysphagia. However, mechanical obstruction associated with coagulation of an enteral formula within the esophagus is extremely rare and may be easily mistaken for ordinary reflux, aspiration, or tube blockage. We report the case of a 69-year-old man with chronic obstructive pulmonary disease (COPD) who received nasogastric tube feeding with RuiNeng enteral nutrition formula, a high-protein, fiber-containing formula, because of impaired consciousness and critical illness. During tube feeding, the patient developed regurgitation of medication, overflow of enteral formula from the oral cavity, marked resistance during aspiration and injection through the nasogastric tube, and withdrawal of a milky-white paste-like substance. Bedside gastroscopy revealed extensive retention of whitish solid coagulated material in the esophageal lumen, causing severe esophageal obstruction. Partial endoscopic removal and fragmentation of the coagulated material were performed, the remaining material was cautiously pushed into the stomach under direct endoscopic visualization, and a jejunal feeding tube was placed under direct endoscopic visualization. Based on the clinical course and published reports, this case was probably associated with suspected impaired opening of the esophagogastric junction, possible nasogastric tube malposition, migration, coiling, or ineffective passage into the stomach with subsequent retention of enteral formula in the esophagus, and coagulation of the high-protein formula in a locally acidic or low-motility environment. Because no objective pH measurement of esophageal contents or the coagulated material was available, these mechanisms should be interpreted as plausible hypotheses rather than directly proven findings. This case highlights that, in critically ill patients receiving high-protein or fiber-containing enteral formulas, abnormal reflux, sudden tube resistance, or milky-white paste-like aspirate should prompt immediate cessation of feeding, reassessment of tube position and esophageal patency, and early bedside endoscopic diagnosis and treatment when necessary.

## Introduction

Enteral nutrition helps maintain gastrointestinal function, improves nutritional intake, and may reduce nutrition-support-related risks in selected critically ill patients. It is an important nutritional support strategy for patients with a functional gastrointestinal tract who cannot meet their nutritional requirements orally, and it is widely used in critically ill, perioperative, oncologic, and dysphagic populations (1–3). Common complications include diarrhea, abdominal distension, vomiting, gastric retention, reflux, aspiration, tube blockage, accidental dislodgement, and tube malposition (4–8). Compared with these common problems, coagulation of enteral formulas within the esophagus, forming concretions or bezoars and causing acute esophageal obstruction, has rarely been reported.

In previous reports, tube-feeding-formula-related esophageal bezoars or concretions have most often occurred in patients in intensive care, patients who were bedridden for prolonged periods, mechanically ventilated patients, and those with nasogastric tube malposition, impaired esophageal motility, gastroesophageal reflux, or exposure to acid-suppressive drugs or sucralfate (9–15). These cases commonly present with difficulty reinserting a nasogastric tube, tube obstruction, oral or nasal reflux of solidified formula, dysphagia, or acute esophageal obstruction, and the diagnosis is usually confirmed by endoscopy. We report a case of acute complete esophageal obstruction associated with intraesophageal coagulation of RuiNeng enteral nutrition formula, aiming to increase clinical awareness of this rare mechanical complication. This case report was prepared with reference to the CARE case-reporting guidelines (16).

## Case description

### Patient information and admission status

A 69-year-old man was admitted on October 25, 2025, at 12:58 because of recurrent cough and sputum production for more than 10 years, accompanied by chest distress and dyspnea for more than 3 years, with worsening symptoms during the preceding week. On November 7, 2025, at 09:57, he was transferred from the general ward of the Department of Respiratory and Critical Care Medicine to the respiratory intensive care unit (RICU) because of aggravated dyspnea. Diagnoses after transfer to the RICU included COPD with acute lower respiratory tract infection, pulmonary encephalopathy, type II respiratory failure, grade 3 hypertension, respiratory acidosis, and pneumothorax.

After admission to the RICU, the patient received intensive nursing care, electrocardiographic monitoring, non-invasive ventilatory support, anti-infective therapy, bronchodilator and antispasmodic therapy, anti-inflammatory treatment, nebulized inhalation, expectorant therapy, and acid suppression. Closed thoracic drainage was performed for pneumothorax. Because of impaired consciousness and critical illness, the patient could not eat orally.

### Enteral nutrition administration

Because the patient was unconscious and could not safely take food orally, a nasogastric tube was inserted after transfer to the RICU according to medical orders. The insertion depth was 60 cm. Before the tube was first used, two staff members used auscultation: a stethoscope was placed over the gastric area, 20 mL of air was rapidly injected through the tube, and a clear air-insufflation sound was heard. The tube tip was therefore judged at the bedside to be in the stomach (17). On November 8, 2025, at 09:56, long-term medical orders were initiated for enteral nutritional support with RuiNeng enteral nutrition formula. During tube feeding, the head of the bed was elevated by 30–45 degrees. RuiNeng is a compound enteral nutrition emulsion (TPF-T; Fresenius Kabi Huarui Pharmaceutical Co., Ltd.); each 500 mL contains 29.3 g protein, 36 g fat, 52 g carbohydrate, 6.5 g dietary fiber, multiple trace elements, and vitamins (18). The patient received 1,000 mL/day of RuiNeng via the nasogastric tube at 50 mL/h. After the initial mention of the brand name, this product is referred to as a high-protein, fiber-enriched enteral formula.

During the approximately 10 days from the initiation of enteral nutrition to the acute event, no early warning signs such as acid reflux, hiccups, increased gastric residual volume, vomiting, aspiration, oral or nasal reflux, or tube obstruction were recorded. The medical and nursing records during tube feeding did not indicate obvious gastrointestinal intolerance or reflux events. At 12:50 on November 17, 2025, when medication was administered through the nasogastric tube, a small amount of medication refluxed into the oral cavity. Two staff members again placed a stethoscope over the gastric area and rapidly injected 20 mL of air through the tube; a clear air-insufflation sound was heard. Approximately 20 g of milky-white paste-like material was then aspirated. At 18:30, before nasogastric administration of oral medications, marked resistance was encountered during both aspiration and injection, and enteral formula was observed overflowing from the patient's mouth. Administration of RuiNeng and oral medications through the nasogastric tube was stopped according to medical orders. At 15:00 on November 17, 2025, bedside bronchoscopy performed by a respiratory therapist, together with imaging findings, showed no abnormal signs of pulmonary choking or aspiration.

### Symptom onset, diagnostic assessment, and endoscopic management

At 12:50 on November 17, 2025, during medication administration through the nasogastric tube, a small amount of medication refluxed into the patient's oral cavity, and approximately 20 g of milky-white paste-like material was aspirated through the tube. At 18:30, reassessment before nasogastric medication administration revealed marked resistance during both aspiration and injection, with enteral formula overflowing from the mouth. Esophageal obstruction was suspected. Administration through the nasogastric tube was stopped, bedside gastroscopy was planned, and jejunal feeding tube placement was considered if necessary.

The patient had undergone orotracheal intubation on November 13, 2025 because of clinical deterioration and remained under continuous analgesia and sedation. His Richmond Agitation-Sedation Scale score was −2. Continuous intravenous infusions included 0.9% sodium chloride 45 mL plus butorphanol injection 5 mg at 5 mL/h and cyclopofol injection 100 mg at 5 mL/h. Before bedside gastroscopy, cyclopofol injection 3 mL was administered intravenously as a bolus, followed by continuous infusion at 5 mL/h. The total volume of cyclopofol used during the procedure was 8 mL (20 mg; specification: 50 mg/20 mL). At 16:30 on November 18, 2025, bedside gastroscopy and jejunal feeding tube placement were performed. After removal of the nasogastric tube, its distal end was found to be obstructed by a white, milky paste.

**Figure 1:**
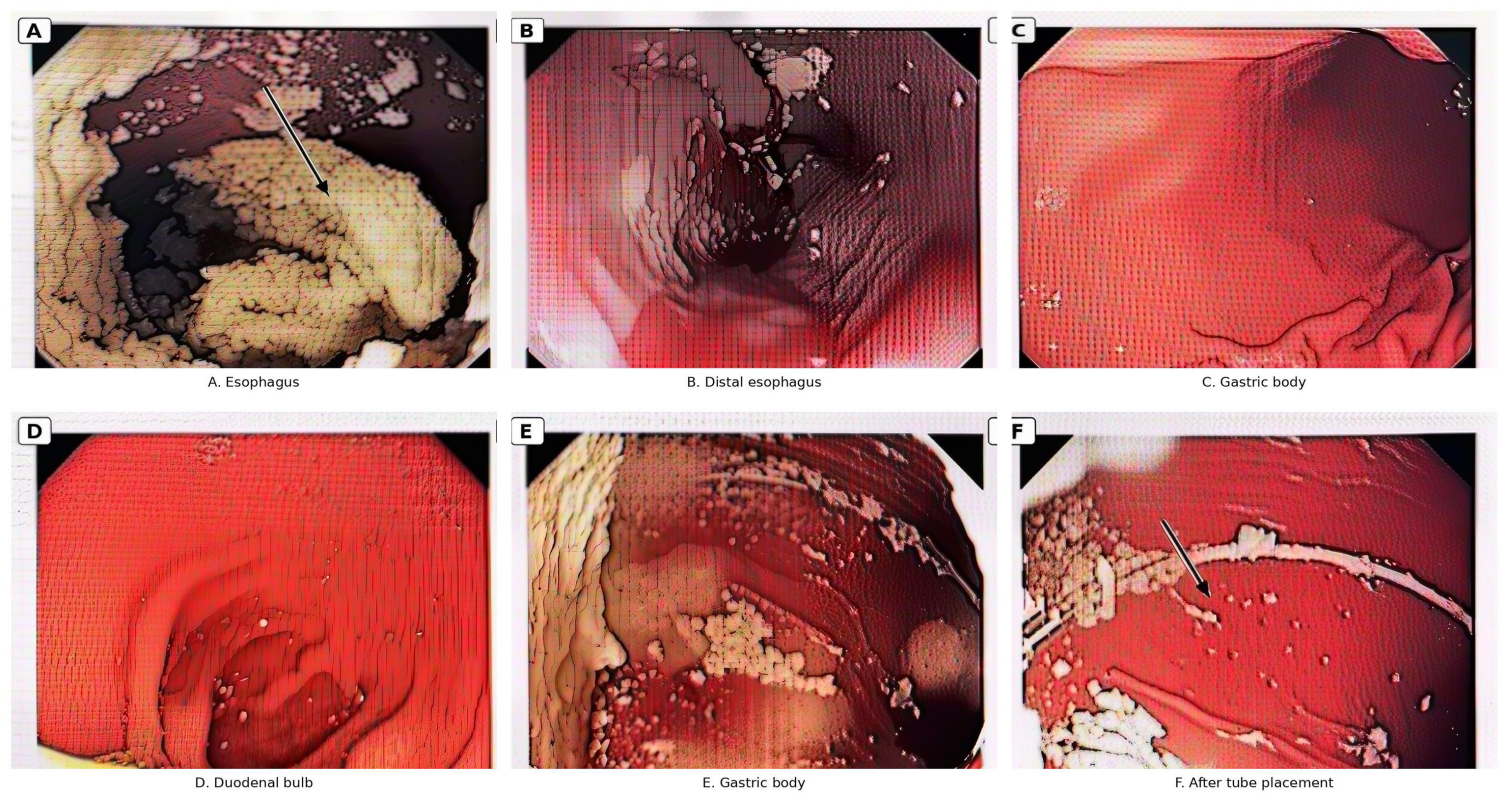
Endoscopic findings and tube placement. **(A)** Large whitish solid/paste-like material occupying the esophageal lumen. **(B)** Distal esophagus after partial fragmentation, with residual adherent material and luminal narrowing. **(C)** Gastric body. **(D)** Duodenal bulb. **(E)** Gastric body during tube manipulation. **(F)** Post-pyloric tube after endoscopic placement.

Endoscopy showed a large amount of white to pale-yellow solid/paste-like coagulated material retained in the esophageal lumen. The material was extensive, involved the whole esophageal segments, had a thick paste-like to solid texture, and markedly impaired endoscopic visualization. The material was not sent for pH measurement, microbiological examination, pathological examination, or compositional analysis. The endoscopic findings suggested impaired opening of the esophagogastric junction, with distal esophageal/cardial narrowing that may have contributed to retention of the enteral formula. Achalasia was considered but could not be confirmed because esophageal manometry and barium esophagography were not performed. However, because esophageal manometry and barium esophagography were not performed, achalasia could not be confirmed. No obvious mass, active bleeding, perforation, extensive necrosis, or deep ulceration was observed. After excluding obvious mass, active bleeding, perforation, extensive necrosis, deep ulceration, and severe mucosal injury, the endoscopist used a snare to remove part of the coagulated material, fragmented most of the residual material, and cautiously pushed the remaining fragments into the gastric cavity under direct endoscopic visualization. The procedure lasted approximately 60 min. Under direct endoscopic visualization, a jejunal feeding tube was gradually advanced into the stomach, grasped at its distal end with biopsy forceps, and advanced into the descending duodenum. The tube tip was confirmed not to be coiled and not to have been withdrawn with the endoscope. Enteral nutrition was temporarily withheld after the procedure and was resumed via the jejunal tube on November 21 (Table 1, Figure 1).

**Table 1:** Main clinical timeline of the case.

Time	Event	Clinical significance
October 25, 2025, 12:58	The patient was admitted because of worsening cough, sputum production, chest distress, and dyspnea.	The patient had severe underlying disease and was at high nutritional risk.
November 7, 2025, 09:57	Transferred to the RICU because of aggravated dyspnea.	Diagnoses included COPD with infection, pulmonary encephalopathy, type II respiratory failure, and pneumothorax.
After November 7, 2025	A nasogastric tube was inserted to a depth of 60 cm.	No radiographic confirmation was performed; bedside judgment was based only on insertion depth and double-person auscultation of an air-insufflation sound. Therefore, tube malposition, migration, or coiling could not be excluded.
From November 8, 2025	RuiNeng 1,000 mL/day was infused through the tube at 50 mL/h, with the head of the bed elevated by 30-45 degrees.	Continuous feeding lasted approximately 10 days. No reflux, hiccups, increased gastric residual volume, or other abnormal findings were recorded. The patient had no bowel movement on November 8–10, one bowel movement per day on November 11–12, two on November 13, one on November 14, and none on November 15–16; stool characteristics, color, and volume were unremarkable.
November 13, 2025, 09:00	Orotracheal intubation and invasive mechanical ventilation were initiated. Continuous analgesia and sedation were given with 0.9% sodium chloride 45 mL plus butorphanol injection 5 mg at 5 mL/h and cyclopofol injection 100 mg at 5 mL/h.	The patient was somnolent and unresponsive; blood gas analysis suggested severe respiratory acidosis.
November 17, 2025, 12:50	A small amount of medication refluxed into the oral cavity during medication administration, and approximately 20 g of milky-white paste-like material was aspirated from the tube.	Tube obstruction, reflux, or tube malposition should prompt immediate reassessment. The patient had two bowel movements on November 17, with unremarkable stool characteristics, color, and volume.
November 17, 2025, 18:30	Marked resistance occurred during aspiration and injection, and enteral formula overflowed from the oral cavity. Administration of enteral formula and medications through the nasogastric tube was stopped, and acute esophageal obstruction, tube obstruction, or tube malposition was suspected.	Acute esophageal obstruction was highly suspected.
November 18, 2025, 16:30	Bedside gastroscopy and jejunal feeding tube placement were performed. After removal of the nasogastric tube, its distal end was found to be obstructed by white milky paste. Gastroscopy revealed a large amount of whitish solid/paste-like material in the esophagus with distal esophageal/proximal cardial narrowing; endoscopic removal, fragmentation, cautious pushing of residual material into the stomach, and jejunal tube placement were performed.	Endoscopy provided both diagnosis and treatment. On November 18–19, stools became relatively loose, suggesting intestinal dysbiosis, and Bifidobacterium was given to regulate intestinal flora.
Postoperative hospitalization	No endoscopy-related bleeding, perforation, or recurrent esophageal obstruction occurred.	The patient had one bowel movement on November 20 and two bowel movements per day on November 21–22; stool characteristics, color, and volume were unremarkable.
November 24, 2025, 00:58	The patient died after progression of underlying respiratory disease and respiratory acidosis leading to respiratory failure; the family declined repeat tracheal intubation, cardiopulmonary resuscitation, defibrillation, and other resuscitative measures.	The fatal outcome was not directly causally related to esophageal obstruction or endoscopic treatment; it was mainly associated with progression of the underlying respiratory disease and the family declined further invasive resuscitative measures.

### Follow-up and outcomes

After endoscopic treatment, the esophageal obstruction was relieved. No procedure-related active bleeding, perforation, acute aspiration during endoscopy, or short-term recurrent esophageal obstruction occurred. Enteral nutrition through the original nasogastric route was suspended. After endoscopic confirmation of the jejunal tube position, nutritional support was planned to resume on November 21, 2025. According to long-term medical orders, 500 mL/day of Baipuli enteral nutrition formula was administered through the jejunal tube at 50 mL/h on November 21, 2025. The patient tolerated this well, without hiccups, reflux, diarrhea, or other abnormalities. Baipuli is a compound enteral nutrition preparation containing water, maltodextrin, hydrolyzed whey protein, vegetable oil, vitamins, minerals, trace elements, and other essential nutrients.

The patient eventually died during hospitalization at 00:58 on November 24, 2025. This fatal outcome was not directly causally related to the esophageal obstruction or endoscopic treatment; it was mainly associated with progression of the underlying respiratory disease and the family's decision to decline further invasive resuscitative measures. On November 21, 2025, tracheal extubation was performed and non-invasive ventilatory support was initiated. Lobeline was administered by intravenous infusion to stimulate the respiratory center, and bedside bronchoscopic sputum suction was planned. During the 3 days after extubation and withdrawal from invasive mechanical ventilation, the patient was unable to maintain adequate spontaneous respiration, and carbon dioxide levels gradually increased. The family declined repeat tracheal intubation and stated that if the condition continued to deteriorate, cardiopulmonary resuscitation, defibrillation, and other resuscitative measures should not be performed. Before death, the patient had no recurrent esophageal obstruction or aspiration, and no adverse events related to the endoscopic intervention were present. No follow-up gastroscopy was performed after jejunal tube placement; therefore, subsequent endoscopic evaluation of esophageal mucosal healing was unavailable.

### Patient perspective

This case report was reviewed and approved by the Ethics Committee of Chengdu Third People's Hospital (Approval No. 2025-S-387). Because the patient had died, written informed consent for publication of the case details and potentially identifiable endoscopic images was obtained from the patient's next of kin. All patient information was anonymized as far as possible.

## Discussion

### Rarity and literature review

Enteral-nutrition-related mechanical obstruction of the esophagus is not a common complication. Previous reports have used terms such as “esophageal bezoar,” “enteral feed bezoar,” “tube-feeding formula concretion,” and “solidification of enteral feed” to describe similar phenomena (10–15). Shared features include critical illness or prolonged bed rest, nasogastric tube placement, impaired esophageal or gastric motility, reflux, tube malposition, prolonged or continuous formula infusion, and exposure to acid-suppressive medications, sucralfate, sedatives, or analgesics.

Compared with previous cases, the present case had several distinctive features. The patient was critically ill with COPD, type II respiratory failure, and multiple comorbidities. He received a high-protein, fiber-containing RuiNeng formula and developed increased tube resistance, aspiration of milky-white paste-like material, and overflow of enteral formula from the oral cavity after approximately 10 days of tube feeding. Endoscopy suggested impaired opening of the esophagogastric junction and distal esophageal/cardial narrowing, which may have contributed to formula retention and coagulation. However, because esophageal manometry or barium esophagography was not performed, achalasia could not be confirmed. The clinical process was consistent with the pattern of an enteral formula retained in the esophagus, followed by coagulation and mechanical obstruction (Table 2).

**Table 2:** Comparison of previously reported enteral-nutrition-related esophageal concretions/bezoars and the present case.

Reference	Patient/background	Suspected risk factors	Diagnosis and management	Lessons relevant to the present case
Irgau et al. (10)	Obstruction associated with tube-feeding formula concretion.	Nasogastric tube, critical illness, and formula coagulation.	Reported concretions of tube-feeding formula within the esophagus.	Liquid enteral formulas may become mechanical obstructive material under specific conditions.
Marcus et al. (11)	A long-term mechanically ventilated patient with difficulty reinserting a tube.	Long-term nasogastric feeding and impaired esophageal clearance.	Gastroscopy showed a long-segment enteral formula concretion; endoscopic fragmentation/removal was performed.	Difficulty reinserting a tube or tube obstruction should raise suspicion for esophageal nutrition bezoar.
Gil-Almagro et al. (12)	Two ICU patients with oral or nasal reflux of solid enteral nutrition.	Prolonged ICU stay, reflux, low motility, and formula-related factors.	Gastroscopy confirmed the diagnosis; enzymatic dissolution was successful.	Oral or nasal reflux of solid material is an important early warning sign.
Degheili et al. (13)	A patient after cardiac arrest with prolonged ventilation and nasogastric tube feeding.	Casein-containing formula, long-term tube feeding, and critical illness.	Imaging/endoscopy identified calcified-appearing material in the distal esophagus.	Casein-based formulas may form persistent concretions in high-risk environments.
Morell et al. (14)	Several critically ill patients with esophageal nutrition bezoars.	Fiber-containing formula, acidic environment, and esophageal accumulation.	Local instillation of 8.4% sodium bicarbonate and *in vitro* experiments supported low-pH-induced coagulation.	Acidic environments have experimental support for promoting coagulation, but pH was not measured in this case and the mechanism should be stated cautiously.
Llobera et al. (15)	Enteral-nutrition-related small-bowel bezoar with literature review.	The review summarized the association between nasogastric tube malposition and esophageal bezoar.	Reviewed nutrition bezoars in esophageal and non-esophageal sites.	Enteral nutrition can form mechanical obstructions at different sites of the gastrointestinal tract.
Present case	A 69-year-old man with COPD and type II respiratory failure who received RuiNeng tube feeding in the RICU for approximately 10 days.	Endoscopy suggested impaired opening of the esophagogastric junction and distal esophageal/cardial narrowing, which may have contributed to formula retention and coagulation. Achalasia was considered but was not confirmed because manometry or barium esophagography was not performed; possible nasogastric tube malposition, migration, coiling, or ineffective passage into the stomach was suspected; continuous low-rate infusion and a high-protein/fiber-containing formula may have contributed. However, achalasia was not confirmed because manometry or barium esophagography was not performed.	Bedside gastroscopy confirmed the diagnosis; partial snare removal, fragmentation, pushing of residual material into the stomach, and jejunal tube placement were performed.	Abnormal reflux, milky-white paste-like aspirate, and increased injection resistance should prompt immediate cessation of feeding, tube-position reassessment, and endoscopic evaluation.

### Potential mechanisms

#### Formula-related factors: high protein, fiber, and possible coagulation in acidic environments

RuiNeng is a high-energy-density, high-protein enteral formula that contains dietary fiber. Previous studies suggest that some casein-containing or high-protein enteral formulas can precipitate, aggregate, or form masses in acidic environments; fiber-containing formulas may increase viscosity and provide a scaffold for solid-structure formation (12, 14). Morell et al. showed *in vitro* that some fiber-containing enteral formulas can form coagulated material when pH decreases, and that such material can be dissolved by sodium bicarbonate (14). However, in the present case, pH was not measured in esophageal secretions, coagulated material, or gastric contents, and compositional analysis of the coagulated material was not performed. Therefore, “acid-induced coagulation of RuiNeng” should be interpreted as a plausible mechanism based on the literature and the clinical course rather than a mechanism directly proven in this patient.

#### Patient- and tube-related factors: distal esophageal/cardial narrowing and suspected tube malposition

Endoscopy suggested distal esophageal/cardial narrowing or impaired opening of the esophagogastric junction, and achalasia could not be excluded. However, because esophageal manometry, barium esophagography, and repeat endoscopy were not performed, the cause remained unconfirmed. Given the sudden oral overflow of formula after prolonged tube feeding, markedly increased tube resistance, obstruction of the removed tube tip by milky-white paste, and absence of a comparable amount of milky-white coagulated material in the stomach (because the tube position was not confirmed radiographically, the exact location of the tube tip during feeding remains uncertain), the nasogastric tube may not have stably passed through the narrowed segment into the stomach, or it may have migrated upward or coiled later, causing long-term retention of formula in the esophageal lumen proximal to the narrowing.

This case also illustrates that reliance on insertion depth, an air-insufflation sound on auscultation, or aspiration alone cannot reliably exclude nasogastric tube malposition or coiling (17). Although this report recommends radiographic confirmation after tube insertion, no imaging confirmation was performed in this patient at the time. The tube position was repeatedly judged by insertion depth and auscultation of an air-insufflation sound. Because the tube tip was close to the cardia, the sound was clearly heard over the gastric region, which may have contributed to delayed recognition of the abnormal tube position.

#### Clinical operational factors: continuous low-rate infusion and prolonged retention

The patient received RuiNeng continuously at a low rate of 50 mL/h for approximately 10 days. If the tube tip was located in the esophagus or near the stenotic segment, continuous low-rate infusion could have allowed gradual accumulation of small amounts of formula in the esophagus. Ineffective esophageal peristaltic clearance would have increased opportunities for mixing with secretions or refluxed gastric acid, temperature reduction, increased viscosity, and local water absorption (19, 20). Compared with a single large-volume infusion error, continuous low-rate delivery may create a more insidious process of progressive accumulation, thickening, coagulation, and obstruction.

### Diagnostic reasoning and differential diagnosis

The initial manifestations were medication reflux, aspiration of milky-white paste-like material, marked resistance during tube aspiration and injection, and oral overflow of formula. These signs may occur with tube blockage, gastric retention, and reflux, but they should also prompt suspicion of tube malposition or esophageal luminal obstruction. Bedside gastroscopy directly demonstrated a large amount of whitish solid/paste-like material with an appearance similar to enteral formula and allowed simultaneous therapeutic intervention. It was therefore central to both diagnosis and management.

The differential diagnosis included the following. First, ordinary food impaction was considered unlikely because the patient had been unable to take food orally for a prolonged period, and the obstructive material was milky-white and paste-like, matching the appearance of the enteral formula. Second, esophageal malignancy was considered less likely because endoscopy showed no cauliflower-like mass, obvious space-occupying lesion, or contact bleeding; however, the presence of distal esophageal/cardial narrowing meant that repeat endoscopy or pathological evaluation would have been useful if the patient's condition had allowed. Third, fungal esophagitis was less likely because the main finding was intraluminal coagulated material; typical pseudomembranous plaques, diffuse mucosal congestion/erosion, and mycological evidence were absent. Fourth, medication bezoar was considered because the patient received medication through the tube, but the obstructive material mainly resembled milky-white nutritional material, with no clear evidence of medication agglomeration or specific drug deposition. Fifth, severe reflux-related obstruction could have contributed to formula coagulation, but reflux alone could not fully explain the prolonged localization of a large amount of nutrition-like coagulated material in the esophagus. Sixth, achalasia or another motility disorder could not be excluded because no history of dysphagia or esophageal manometry data was available; achalasia is usually confirmed by high-resolution esophageal manometry, and barium esophagography can provide supportive evidence (21).

### Management experience

When acute esophageal obstruction associated with coagulated enteral formula is suspected, infusion through the original tube should be stopped immediately to avoid continued injection, which may increase the risks of aspiration, esophageal dilation, or perforation. Bedside endoscopy is suitable for critically ill patients in whom transport carries substantial risk. It allows direct assessment of the obstruction site, the nature of the coagulated material, the degree of mucosal injury, and the presence of stenosis, while also enabling fragmentation, removal, or cautious pushing of residual material into the stomach after endoscopic assessment excluded obvious perforation, active bleeding, extensive necrosis, deep ulceration, or severe mucosal injury using a snare, retrieval net, or foreign-body forceps. In this case, partial removal, fragmentation of residual material, and pushing of fragments into the stomach were performed endoscopically, followed by direct endoscopic placement of a jejunal feeding tube to avoid repeated blind nasogastric tube insertion. This approach addressed both obstruction relief and subsequent nutritional support, but risks such as aspiration during fragmentation, mucosal laceration, bleeding, perforation, and recurrent obstruction from residual coagulated material should be kept in mind.

### Prevention and management recommendations

#### Tube-position verification

After placement of a nasogastric or nasoenteric tube, the tube position should be verified according to institutional protocols. For critically ill or unconscious patients, patients with esophageal stenosis or motility disorders, those with a history of esophageal disease, and those in whom tube reinsertion is difficult, radiography, fluoroscopy, or endoscopic guidance should be prioritized to confirm that the tube tip is in the intended position (17). For patients requiring long-term nutritional support who have high-risk esophageal features, direct nasoenteric tube placement under endoscopic or fluoroscopic guidance, or alternative routes such as percutaneous gastrostomy or jejunostomy, should be considered.

#### Optimization of infusion protocol

In high-risk patients, infusion rate and method should be balanced against nutritional targets, gastrointestinal tolerance, and aspiration risk. If the tube tip is clearly located in the stomach or jejunum, continuous, cyclic, intermittent, or bolus feeding can be selected according to the patient's condition (19). However, when abnormal resistance, oral or nasal reflux, or milky-white paste-like aspirate occurs, feeding should be stopped immediately and the tube position and esophageal patency reassessed; these warning signs should not be addressed merely by flushing or forceful injection. During and after feeding, the head of the bed should remain elevated by at least 30 degrees, and reflux, vomiting, gastric residual volume, and respiratory symptoms should be monitored.

#### Tube flushing and medication compatibility management

According to nutrition-support guidelines and tube-management practice, the tube should be adequately flushed with warm water before and after enteral formula infusion and before and after medication administration. In adults, a commonly used flush volume is approximately 30 mL; however, this should be individualized according to fluid restriction, renal function, cardiac function, and considerations in children or low-body-weight patients (1, 22–24). Unless compatibility is clearly supported, enteral formulas should not be mixed or co-infused with medications, particularly acidic liquids or drugs that may interact with proteins or fiber. Medications should be administered one at a time, with flushing before and after each drug, and multiple tablets should not be crushed together and mixed with enteral formula (23, 24).

#### Nursing monitoring and early-warning education

Nursing staff should recognize abnormal reflux, oral or nasal overflow of formula, aspiration of milky-white paste-like material, sudden tube resistance, and difficulty reinserting a tube as warning signs of intraesophageal formula coagulation or tube malposition. If these signs occur, feeding should be stopped, the physician notified, tube position reassessed, and endoscopic evaluation initiated early when necessary.

### Limitations

This case has several limitations. First, this is a single case report, and the findings cannot be generalized without further clinical observations. Second, radiographic confirmation was not performed after the initial nasogastric tube placement or during subsequent feeding. Therefore, the exact position of the tube and the timing of any possible malposition, migration, or coiling could not be determined. Third, pH was not measured in the esophageal lumen, coagulated material, or gastric contents, and compositional analysis of the coagulated material was not performed. Therefore, the proposed mechanisms, including acid-related coagulation, temperature change, increased viscosity, and formula retention, remain inferential. Fourth, the cause of distal esophageal/cardial narrowing or suspected impaired opening of the esophagogastric junction was not confirmed because esophageal manometry, barium esophagography, pathological examination, and follow-up endoscopy were not performed. Therefore, achalasia or another esophageal motility disorder could not be diagnosed definitively. Fifth, although no immediate endoscopy-related bleeding, perforation, aspiration, or recurrent obstruction was observed, long-term follow-up was unavailable because the patient died from progression of the underlying respiratory disease. Therefore, long-term esophageal mucosal healing and the risk of recurrent obstruction could not be evaluated.

## Conclusion

Nasogastric tube feeding is not a completely risk-free routine procedure. This case suggests that in critically ill patients with possible distal esophageal/cardial narrowing or impaired opening of the esophagogastric junction, suspected nasogastric tube malposition, and prolonged low-rate infusion of a high-protein, fiber-containing enteral formula, the formula may be retained and coagulate in the esophagus, potentially resulting in severe esophageal obstruction. When increased tube resistance, milky-white paste-like aspirate, oral or nasal reflux, or difficulty reinserting a tube occurs, feeding should be stopped immediately, tube position and esophageal patency should be reassessed, and bedside endoscopic diagnosis and treatment should be considered early. Standardized tube-position verification, timely recognition of warning signs, and individualized feeding-route selection for high-risk patients are essential for preventing this rare but severe complication.

## Data Availability

The original contributions presented in the study are included in the article/supplementary material, further inquiries can be directed to the corresponding author.
